# Supplementary material for: Visualising the Emerging Platform of Using Microalgae as a Sustainable Bio-Factory for Healthy Lipid Production through Biocompatible AIE Probes
Source: Biosensors (Basel). 2022 Mar 31;12(4):208. doi: 10.3390/bios12040208 (PMC9029145; doi:10.3390/bios12040208)
Supplement: Supplementary file 1 [file biosensors-12-00208-s001.zip › biosensors-1639947-supplementary.pdf]

biosensors

Supporting Information

Visualising the Emerging Platform of Using Microalgae as a Sustainable Bio-Factory for Healthy Lipid Production through Biocompatible AIE Probes

AHM Mohsinul Reza [1,2], Sharmin Ferdewsi Rakhi [1,2], Xiaochen Zhu [1,2], Youhong Tang [1,2] and Jianguang Qin [1,*]

[1] College of Science and Engineering, Flinders University, SA 5001, Australia; ahmmohsinul.reza@flinders.edu.au (A.M.R.); rakh0007@flinders.edu.au (S.F.R.); zhu0351@flinders.edu.au (X.Z.); youhong.tang@flinders.edu.au (Y.T.)
[2] Institute for NanoScale Science and Technology, College of Science and Engineering, Flinders University, SA 5001, Australia
* Correspondence: jian.qin@flinders.edu.au; Tel.: +61-8-82013045

Autofluorescence properties of C. reinhardtii

The presence of chlorophyll and different natural pigments causes strong autofluorescence in microalgae that often create interference during fluorescent staining of target molecules. To select the appropriate phosphorescent probes for studying the lipid and H_2O_2, spectrophotometric analysis of the autofluorescence spectral profile of live *C. reinhardtii* cells was done to minimize the background noise in this study. Algal cells excited at 350 nm emitted weak fluorescence at around 400 nm (Figure S1a), whereas excitation at 405 nm resulted in two emission peaks at around 470 nm and 685 nm (Figure S1b). A single emission peak at 685 nm was observed, while cells were excited at 488 nm. The cause of the fluorescence at around 400 nm was unclear, while that of around 470 nm was assumed due to the number of redox ratios (NAD(P)H/FAD) [1]. The emission peak at 685 nm was due to the autofluorescence from chlorophyll. As shown in Figure S1c, excitation at 488 nm resulted in a single peak with maximum fluorescence from chlorophyll.

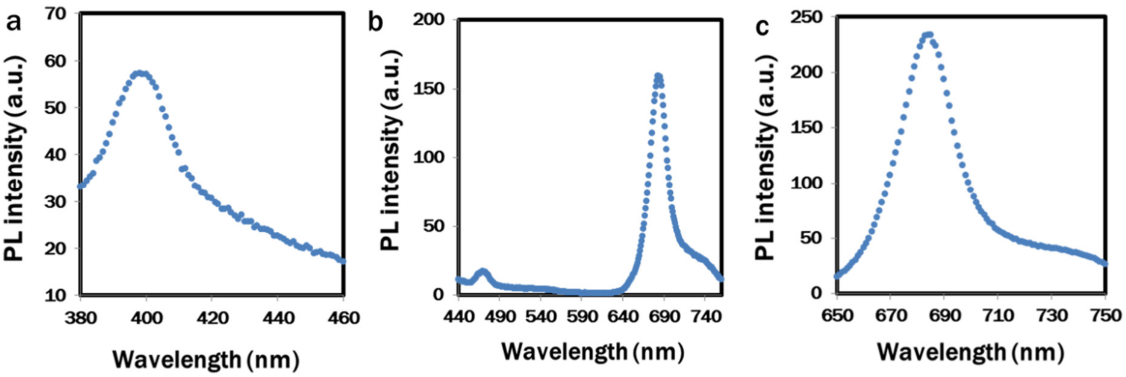

Figure S1. Autofluorescence of *Chlamydomonas reinhardtii* excited at different wavelength (a) λ_{ex}: 350 nm; (b) λ_{ex}: 405 nm; (c) λ_{ex}: 488 nm.

Algal growth and acquisition of probe concentration

Prior to the lipid analysis, the effects of 2-DPAN on growth of *C. reinhardtii*, fluorescence intensity of 2-DPAN at different concentrations, and at different time intervals were monitored. Compared with the control group, no significant difference was found in the algal growth pattern even at a very high concentration of 100 μM 2-DPAN exposure (Figure S2a). However, an increase in the fluorescence intensity was observed while cells were

treated with 20 μM 2-DPAN in comparison to that of the 5 μM and 10 μM (Figure S2b). Since the differences between the fluorescence intensity of 20 μM and 30 μM were very insignificant, 20 μM was used to label the LDs in this experiment. While incubating the cells at different time intervals, the fluorescence intensity was slightly lower in 10 min incubation than that of the 30 min (Figure S2c). As no significant difference was observed between the 30 min and 60 min incubation, it was assumed that 2-DPAN could completely label lipid drops in *C. reinhardtii* cells within 30 min of the incubation period.

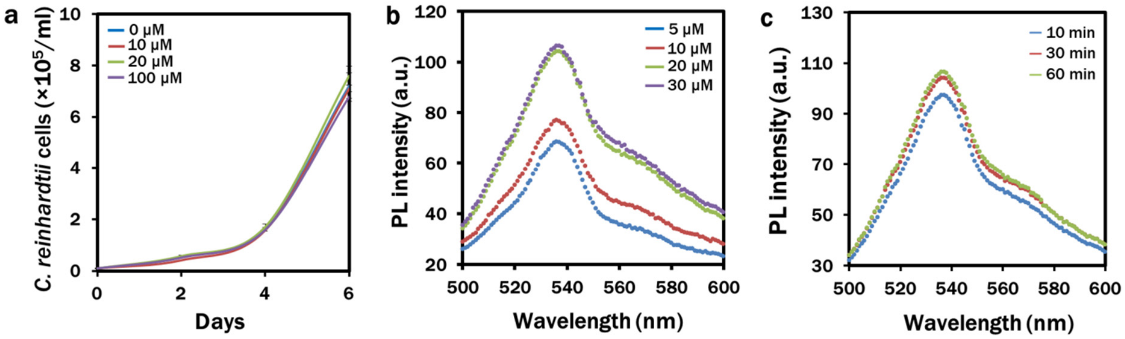

Figure S2. Growth of *Chlamydomonas reinhardtii* and acquisition of AIEgen concentration. (a) algal growth; (b) fluorescence intensity at different concentrations of 2-DPAN exposure; (c) fluorescence spectra of 2-DPAN (20 μM) in *C. reinhardtii* cells at different time intervals. For growth analysis, *C. reinhardtii* cells were cultured in MBL medium; (b, c) to determine the fluorescence intensities, *C. reinhardtii* cells (10^6 cells/mL) of Treatment 4 (MCM, (-) nitrogen, (-) calcium, (+) sodium acetate (2.0 g/L)) was used.

Wash free imaging technique with 2-DPAN

The sample preparation techniques among the AIE probe, 2-DPAN and the traditional probe BODIPY™ 505/515 were compared (Figure S3), and the wash free samples' fluorescence intensity was analyzed spectrophotometrically (Figure S4). Compared to the BODIPY dye, sample preparation with 2-DPAN required fewer steps that excluded the centrifugation and washing steps.

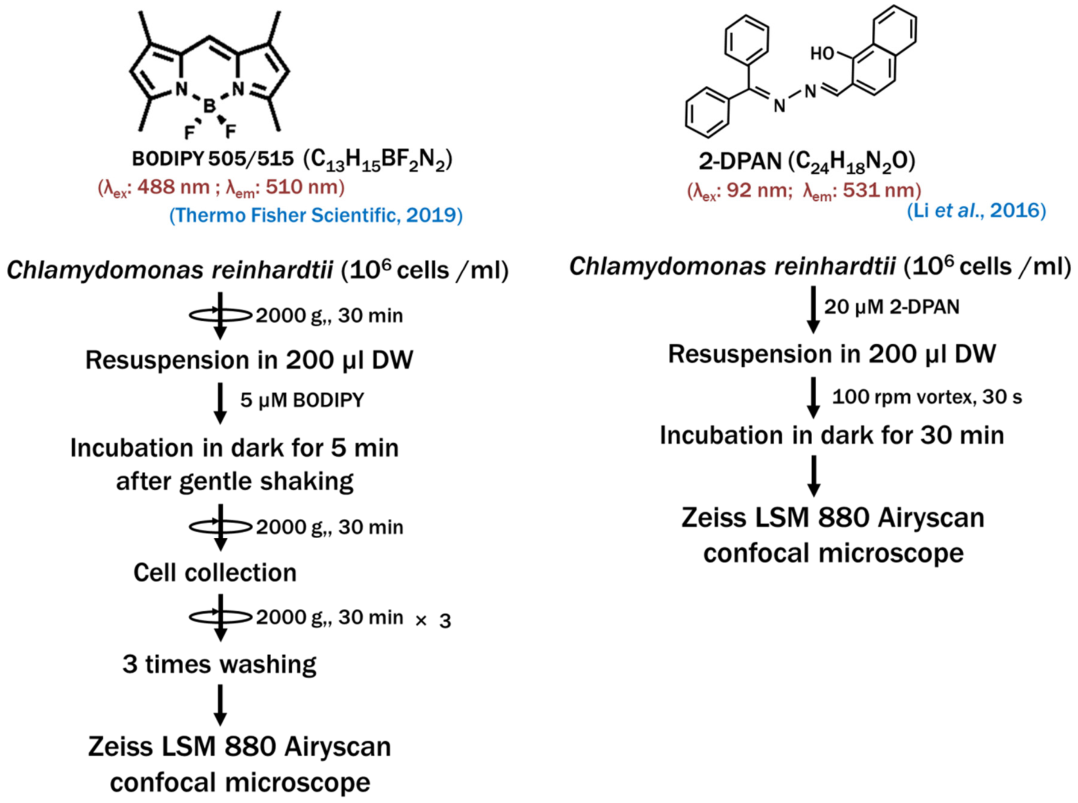

Figure S3. Flow-diagram of sample preparation steps with BODIPY 505/515 and 2-DPAN.

Spectrophotometric results suggested that without washing fluorescence intensity of the BODIPY dye reached outside the meaningful range of the spectrometer (Figure S4a). In contrast, there was almost no difference in the PL intensities between the wash and wash-free samples with 2-DPAN (Figs. S4b). Furthermore, confocal images of the wash free BODIPY samples could not distinguish the chlorophyll from the lipid as no autofluorescence could be detected in the red channel, which was due to the photobleaching of the samples (Figure S4c- S4f).

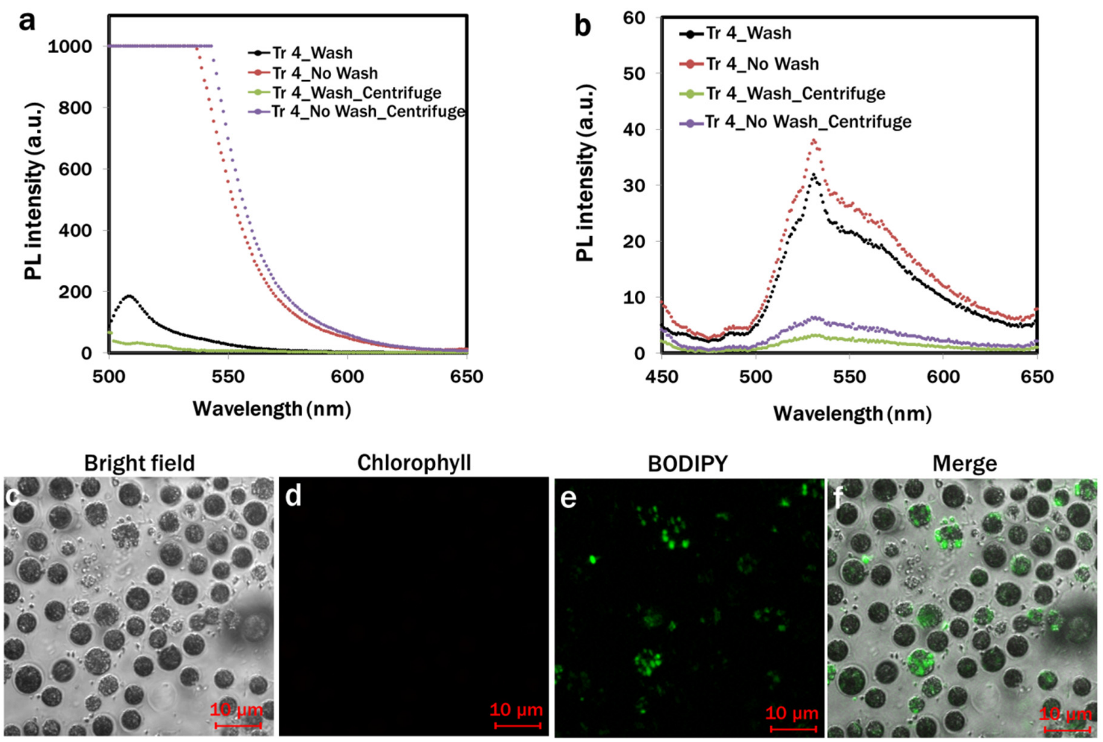

Figure S4. Analysis of the *Chlamydomonas reinhardtii* cells prepared with and without washing steps. Spectrophotometric analysis of the samples prepared with (**a**) BODIPY™ 505/515 (10 μM; incubation- 5 min; λ_{ex}: 488 nm, λ_{em}: 685-758 nm); (**b**) 2-DPAN (20 μM; incubation-30 min; λ_{ex}: 488 nm, λ_{em}: 570-650 nm). (**c-f**) Confocal images of the *C. reinhardtii* cells prepared without washing steps incubated with BODIPY dye. Lipid induced *C. reinhardtii* cells from Treatment 4 (MBL medium, (-) N_2, (+) Sodium Acetate (2.0 g/L), (-) Ca^{2+}) (24 h light condition) were analyzed. Images were taken with Zeiss LSM 880 Airyscan confocal microscope.

Growth of Chlamydomonas reinhardtii in different concentration of TPE-BO

The effects of different concentrations of H_2O_2-specific AIE probe, TPE-BO on *C. reinhardtii* growth were monitored. Compared with the control group, no significant difference was found in the growth pattern of algae even at a very high concentration of 100 μM of TPE-BO exposure (Figure S5).

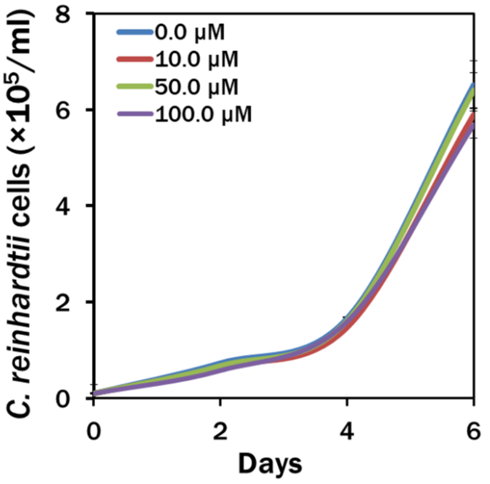

Figure S5. Growth of *Chlamydomonas reinhardtii* in different concentration of TPE-BO.

Growth of Chlamydomonas reinhardtii in different concentration of H_2O_2

We studied the effects of direct H_2O_2 supplementation on the growth of *C. reinhardtii*. The concentration of H_2O_2 was found very critical for cells as almost no change in algal growth was observed until 0.6 mM of H_2O_2 exposure, while a slight increase in H_2O_2 by 0.2 mM to 0.8 mM showed 60% inhibition in cell growth (Figure S6a). Cells did not survive above 1.0 mM H_2O_2 (data are not presented). During monitoring the cells exposed to 0.4 mM (Figure S6b) and 0.6 mM (Figure S6c) H_2O_2, it was obvious that the utilization of H_2O_2 also increased with growth and showed a declining trend during the exponential growth period (Figure S6b and S6c).

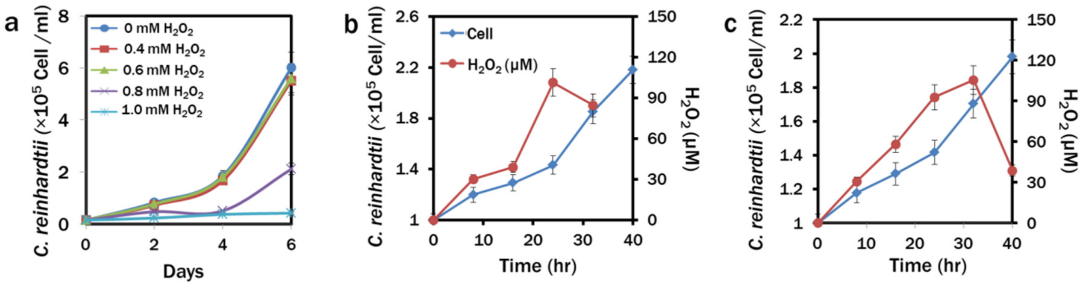

Figure S6. Growth and utilization of H_2O_2 by *C. reinhardtii* cultured in H_2O_2 supplemented MBL medium. Growth of *C. reinhardtii* in different concentration of H_2O_2 (**a**). Utilization of H_2O_2 by the cells grown in 0.4 mM H_2O_2 (**b**) and 0.6 mM H_2O_2 (**c**). Data represented as mean ± SE, n = 3.

Flow cytometric analysis of lipid during H_2O_2 supplementation

The flow cytometric analysis suggested that H_2O_2 supplementation induced lipid in *C. reinhardtii*. From the cytograms of BODIPY and 2-DPAN labelled cells, maximum lipid accumulation was observed in 0.6 mM H_2O_2 supplementation (Figure S7c, S7f, and S7i), followed by the 0.4 mM H_2O_2 (Figure S7b, S7e, and S7i). Control (0.0 mM H_2O_2) group (Figure S7a, S7d, and S7i) showed the lowest amount of lipid accumulation in *C. reinhardtii*

cells. More obese cells due to the lipid accumulation were also apparent from the more significant site scattering of the H_2O_2 supplemented cells (Figure S7b, S7c, S7e, and S7f).

In compare to the control (0.0 mM H_2O_2) group (Figure S7a, S7d, and S7i), Cytograms of BODIPY and 2-DPAN labelled cells showed maximum lipid accumulation in 0.6 mM H_2O_2 supplementation (Figure S7c, S7f, and S7i) that was followed by the 0.4 mM H_2O_2 (Figure S7b, S7e, and S7i). Additionally, more obese cells due to the lipid accumulation were also apparent from the greater side scattering of the H_2O_2 supplemented cells (Figure S7b, S7c, S7e, and S7f) [2]. For further clarification, imaging of the 2-DPAN labelled *C. reinhardtii* cells was done with confocal microscopy.

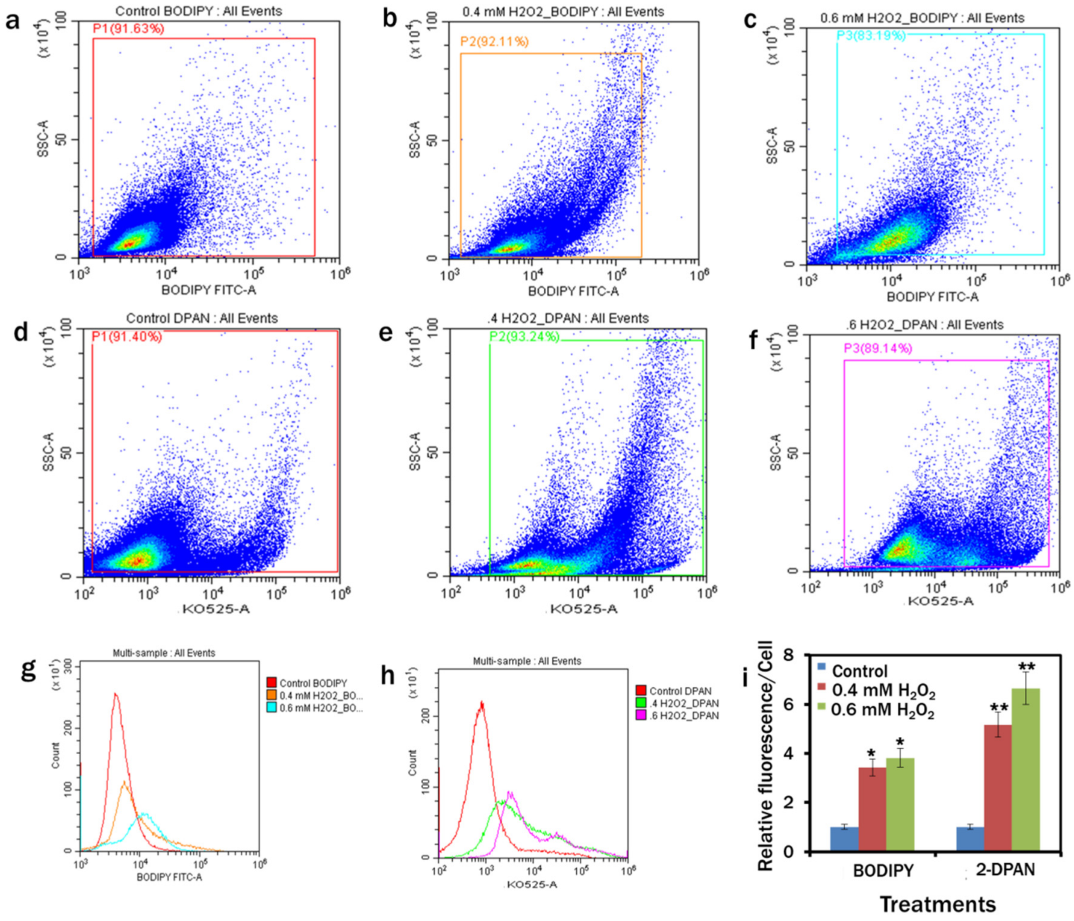

Figure S7. Flow cytometric analysis of lipid accumulation in H_2O_2 treated *Chlamydomonas reinhardtii*. Lipids were labelled with BODIPY™ 505/515 and AIE probe, 2-DPAN. (**a-c**) Flow cytogram of FITC-A vs. SSC-A for BODIPY fluorescence in different treatments; (**d-f**) Flow cytogram of KO525-A vs. SSC-A for 2-DPAN fluorescence. Cells were cultured in 0.0 mM (a, d), 0.4 mM (b, e) and 0.6 mM (c, f) H_2O_2 supplemented MBL medium. Histogram of BODIPY™ 505/515 (**g**) and 2-DPAN (**h**) fluorescence for cells. (i) Relative fluorescence of BODIPY™ 505/515 and 2-DPAN/cell for different treatments. Values are relative to the control condition (0.0 mM H_2O_2). Averages shown as mean ± SE; $*P < 0.05$; $**P < 0.01$. All plots are in a logarithmic scale for both axes.

Fatty acid analysis

Supplementation of H_2O_2 in the MBL medium almost increased the TFA content by two fold to ~13% than that of the Treatment 1 (MBL medium). Among the nutrient altered conditions, maximum amount of TFA (~11% of DW) was found in the cells of Treatment 3 (MBL, (-) N_2, (-) Ca^{2+}) and Treatment 4 (MBL, (-) N_2, (-) Ca^{2+}, (+) sodium acetate (2.0 g/L)) that was followed by the 8.4% TFA content of Treatment 2 (MBL, (-) N_2) (Figure S8).

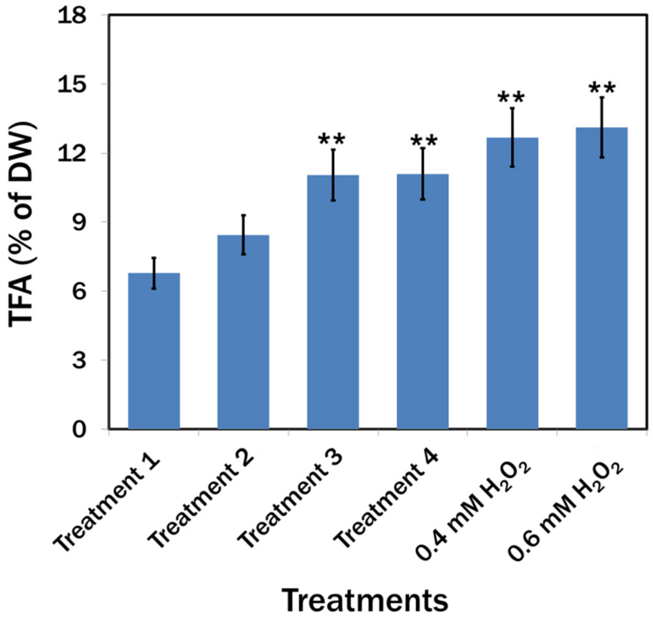

Figure S8. Total fatty acid contents in different treatments. Cells were cultured in Treatment 1: modified Woods Hole (MBL) medium; Treatment 2: MBL, (-) N_2; Treatment 3: MBL, (-) N_2, (-) Ca^{2+}; Treatment 4: MBL, (-) N_2, (-) Ca^{2+}, (+) sodium acetate (2.0 g/L); 0.4 mM and 0.6 mM H_2O_2 supplemented MBL medium (all the treatments were in 24 h light condition). Data represented as Mean ± SE, n = 3, $*P < 0.05$; $**P < 0.01$.

Reference

[1] Wu, Y.; Qu, J.Y. Autofluorescence spectroscopy of epithelial tissues. *J. Biomed. Opt.* **2006**, *11*, 054023. https://doi.org/10.1117/1.2362741.
